# The Presence of Denitrifiers In Bacterial Communities of Urban Stormwater Best Management Practices (BMPs)

**DOI:** 10.1007/s00267-021-01529-z

**Published:** 2021-12-03

**Authors:** Natalie C. Hall, Masoumeh Sikaroodi, Dianna Hogan, R. Christian Jones, Patrick M. Gillevet

**Affiliations:** 1 U.S. Geological Survey, Florence Bascom Geoscience Center, Reston, VA USA; 2 Department of Biology, George Mason University, Manassas, VA USA; 3 Microbiome Analysis Center (MBAC), Manassas, VA USA; 4 U.S. Geological Survey, Deputy Regional Director for Science, Southeast Region, Reston, VA USA; 5 Department of Environmental Science and Policy, George Mason University, Fairfax, VA USA; 6 Potomac Environmental Research and Education Center (PEREC), George Mason University, Woodbridge, VA USA

**Keywords:** Urban stormwater, Best Management Practice (BMP), Land use, Urbanization, Impervious cover, Bacterial denitrifiers

## Abstract

Stormwater best management practices (BMPs) are engineered structures that attempt to mitigate the impacts of stormwater, which can include nitrogen inputs from the surrounding drainage area. The goal of this study was to assess bacterial community composition in different types of stormwater BMP soils to establish whether a particular BMP type harbors more denitrification potential. Soil sampling took place over the summer of 2015 following precipitation events. Soils were sampled from four bioretention facilities, four dry ponds, four surface sand filters, and one dry swale. 16S rRNA gene analysis of extracted DNA and RNA amplicons indicated high bacterial diversity in the soils of all BMP types sampled. An abundance of denitrifiers was also indicated in the extracted DNA using presence/absence of *nirS, nirK*, and *nosZ* denitrification genes. BMP soil bacterial communities were impacted by the surrounding soil physiochemistry. Based on the identification of a metabolically-active community of denitrifiers, this study has indicated that denitrification could potentially occur under appropriate conditions in all types of BMP sampled, including surface sand filters that are often viewed as providing low potential for denitrification. The carbon content of incoming stormwater could be providing bacterial communities with denitrification conditions. The findings of this study are especially relevant for land managers in watersheds with legacy nitrogen from former agricultural land use.

## Introduction

Urbanization is associated with an increase in impervious cover as land use changes from forest, agriculture, and other forms of more highly vegetated land to residential and commercial development. Impervious surface cover leads to an increase in runoff velocity, runoff volume, and peak discharge rate (Arnold and GIbbons 1996). Stormwater runoff transports sediments and other pollutants such as nutrients into receiving water bodies, which can cause a host of problems for urban streams including a decrease in dissolved oxygen (O_2_) levels and eutrophication (Vitousek et al. 1997; Howarth et al. 2002; Rabalais 2002; Wolfe and Patz 2002; Walsh et al. 2005). These conditions have collectively been termed “the urban stream syndrome” (Walsh et al. 2005). The United States (U.S.) Environmental Protection Agency (EPA) lists urban-related stormwater runoff as a major pollutant source (U.S.EPA 2017).

Urban stormwater best management practices (BMPs) are engineered solutions to manage stormwater runoff. BMPs can be placed in series, referred to as treatment trains, to maximize their infiltration and treatment impact (Bastien et al. 2010; Fletcher et al. 2015; Koch et al. 2014). Low impact development or green infrastructure design uses BMPs to mitigate the effects of impervious surface cover with the use of stormwater infrastructure in a way that attempts to mimic natural processes and promotes infiltration and evapotranspiration. This may provide enhanced stormwater treatment in urban and suburban areas (Liu et al. 2014; Fletcher et al. 2015). Understanding the ability of urban BMPs to remove nitrogen from stormwater is critical to optimizing the types of BMPs installed in urban areas to meet nutrient reduction goals. Both the type and density of urban development and stormwater management facilities can influence nutrient transport to streams. Examples include elevated stream baseflow nitrogen concentrations in areas with a history of agriculture (Hopkins et al. 2017), and the removal of more nitrogen when stormwater facilities are distributed on the landscape rather than centralized (for example, isolated ponds) (Sparkman et al. 2017). Stormwater BMP functions can include stormwater detention, conveyance, infiltration, and treatment. Each BMP type has a different soil and media composition depending on designed function.

Denitrification is an anaerobic respiration process whereby nitrite (NO_2_^−^) and nitrate (NO_3_^−^) are sequentially reduced to nitrogen gas (N_2_) via the intermediates nitric oxide (NO) and nitrous oxide (N_2_O) (Zumft 1997; Van Spanning et al. 2007). The process is mediated by a diverse group of microorganisms under low O_2_ or anaerobic conditions (Zumft 1997). The enzymes involved in the reduction of NO_3_^−^ to N_2_ gas (nitrate reductase, nitrite reductase, NO reductase, N_2_O reductase) are encoded by specific genes, namely, *nar*/*nap* (NO_3_^−^ respiration), *nir (*NO_2_^−^ respiration)*, nor* (NO respiration), and *nos* (N_2_O respiration) (Philippot and Hallin 2005). NO_3_^−^ reductase can be either a multiheme enzyme (cytochrome *cd*_*1*_) or a copper-containing enzyme and the genes coding for these enzymes are *nirS* and *nirK* respectively (Zumft 1997). The reduction of N_2_O to N_2_ is the final reaction of the denitrification process and is catalyzed by N_2_O reductase, encoded by the *nosZ* gene, which is missing in many partial denitrifiers with truncated denitrification pathways (Braker et al. 2012) and can result in the release of N_2_O, a known greenhouse gas (Henry et al. 2006; Ligi et al. 2014). The abundance of denitrification genes is recognized as an indicator of denitrification activity (Hallin et al. 2015) and their detection has been applied to numerous soil studies (Rösch et al. 2002; Braker et al. 2012; Henry et al. 2006).

Microbial activity can be related to surrounding soil conditions and studies have indicated that each step of the denitrification process is driven by various soil variables, including pH, water content, temperature, organic carbon (C) concentration, soil texture, NO_3_^−^ and O_2_ availability (Craswell 1978; Knowles 1982; Tiedje et al. 1982; Groffman and Tiedje 1989; Murray et al. 1989; Wallenstein et al. 2006; Perryman et al. 2011; Hallin et al. 2015).

While stormwater BMPs are generally not designed to provide microbially-facilitated denitrification per se, they may offer this ecosystem service as an additional benefit. Denitrification can be encouraged by the intermittent wetting and drying of soils as water levels (and soil O_2_ levels) inside BMPs fluctuate between storm events (Perryman et al. 2011). Urban stormwater BMPs may create opportunities for denitrification due to the physiochemical properties of the incoming stormwater which infiltrates BMP soils. Decomposition and nitrification inside BMPs can also increase the amount of carbon and NO_3_^−^ available to denitrifiers (Bettez and Groffman 2012). Denitrification has been shown to occur in stormwater bioretention facilities (BFs) (Chen et al. 2013), but less attention has been paid to dry ponds (DPs) and surface sand filters (SSFs).

The aims of this study were to (1) assess differences in bacterial communities by BMP type, (2) determine whether these communities were impacted by the surrounding soil physiochemical properties, and (3) assess whether the bacterial communities in each BMP type were active and capable of denitrification.

## Methods

### Sampling

**Fig. 1:**
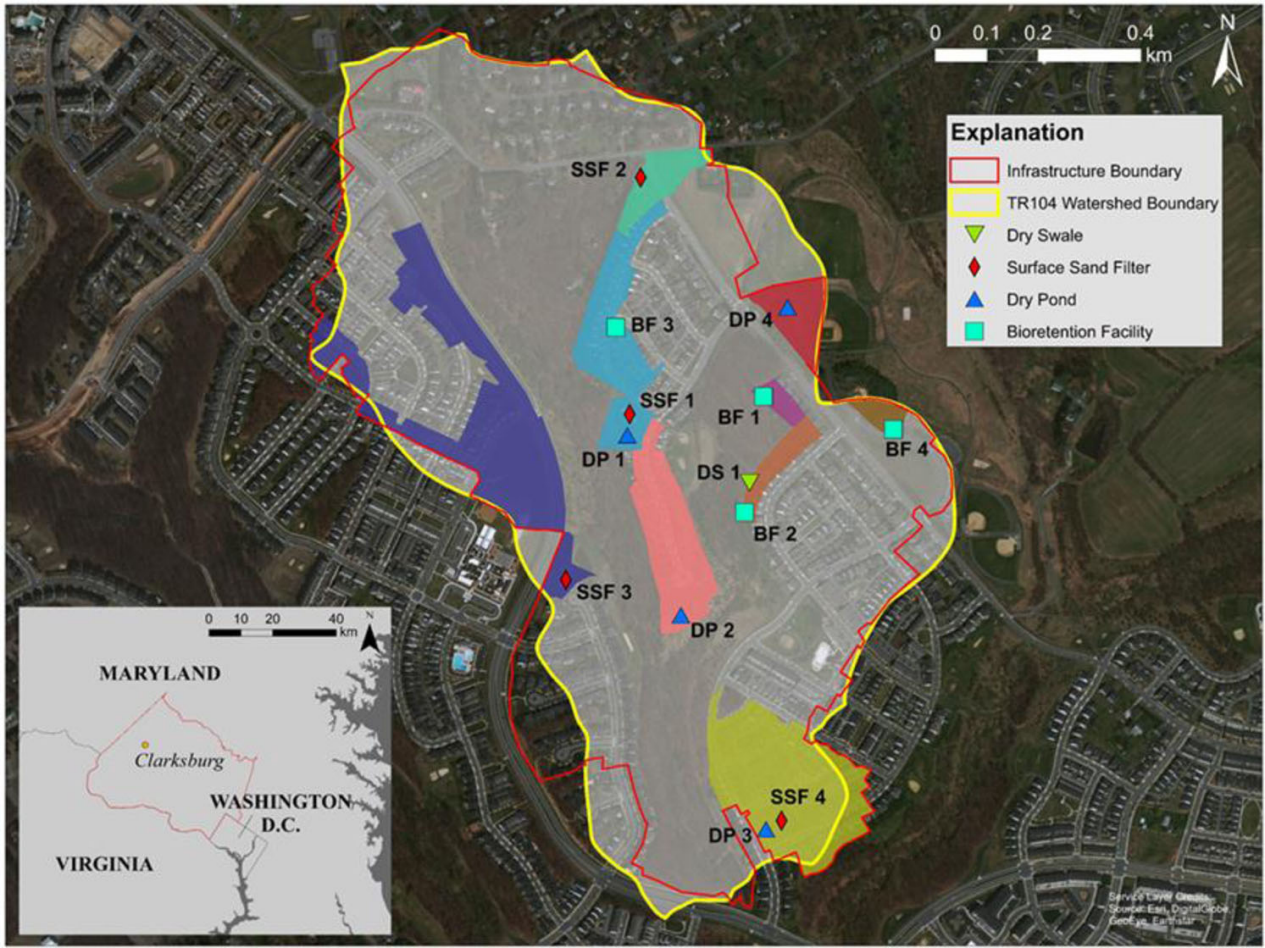
Sampling location and BMP sampling sites in Tributary 104 (TR104), Clarksburg, MD. Drainage areas for each sampled BMP are shaded in color. Sample locations are marked with icons by BMP type. Watershed boundaries indicate above-ground and below-ground stormwater infrastructure

Sampling took place in the Tributary 104 (TR104) watershed in Clarksburg, MD, located in Montgomery County (Fig. 1). The watershed is within the Clarksburg Special Protection Area, an area designated as having high-quality streams and where more stringent regulations are required for new development to protect streams (Montgomery County Department of Environmental Protection 2020). TR104 is a tributary of Little Seneca Creek, and is a developed watershed with distributed infiltration-focused BMPs and impervious cover of 34% (Woznicki and Hopkins 2019). The watershed was converted from predominantly agricultural and forested land cover to suburban development between 2004 and 2010 (Hopkins et al. 2020) (See Table 1 for details of drainage area and impervious cover in sampled BMPs). During development, BMPs were placed in treatment trains across the watershed, with the intention of having stormwater flow from one to the next prior to emptying into the stream. While the effectiveness of BMP treatment train length (i.e., the number of BMPs in a particular treatment train) was not evaluated in this study, concentrations of stream nutrients can be impacted by larger numbers of BMPs, especially when hydrologically connected in series (Holmes et al. 2016).

**Table 1:** Drainage areas and impervious cover (Hopkins et al. 2020) in sampled BMPs

BMP name	Montgomery county name	Drainage area (acres)	Impervious cover (acres)	Impervious cover (%)	Latitude (DD)	Longitude (DD)
BF 1	Bioretention Facility 1	1.09	0.19	17.82	39.240218	−77.255012
BF 2	Bioretention Facility 2	2.30	0.54	23.66	39.238191	−77.255435
BF 3	Bioretention Facility 30	1.95	1.23	63.22	39.241417	−77.258369
BF 4	Bioretention Facility X	1.18	0.37	31.65	39.239646	−77.252108
DP 1	Dry Pond 1	9.04	3.94	43.54	39.239503	−77.258077
DP 2	Dry Pond 2	26.43	12.86	48.67	39.236405	−77.256904
DP 3	Dry Pond 6	16.81	9.88	58.79	39.232666	−77.254931
DP 4	Pond X	3.61	1.17	32.40	39.241713	−77.254548
SSF 1	Surface Sand Filter 1	7.73	3.92	50.65	39.239901	−77.258042
SSF 2	Surface Sand Filter W-SSF13	3.19	0.89	27.89	39.244044	−77.257835
SSF 3	Surface Sand Filter C	23.05	9.52	41.32	39.236993	−77.259481
SSF 4	Surface Sand Filter 6	15.91	9.77	61.40	39.232796	−77.254622
DS 1	Dry Swale (DS) BF02	1.80	0.45	25.14	39.238701	−77.255336

Soils were sampled in 13 BMPs namely four BFs, four DPs, four SSFs, and one dry swale (DS). Further sampling of the DS was prevented by an impenetrable layer within the BMP and the single DS core was not used for statistical analysis. BFs are basins planted with water tolerant grasses, shrubs, and small trees. Stormwater fills the basin for a short period of time, before soaking into the soil. BFs emphasize infiltration, hydraulic control, and water quality. DPs collect stormwater and detain it temporarily (hydraulic control), allowing pollutants to settle. The DPs in this study have grass, water tolerant plants, and other vegetation on the bottom and sides of the structure. DPs emphasize the detention and slowing of stormwater runoff. SSFs are depressions filled with sand and pebbles (mineral media). They are designed to filter stormwater through the media to remove pollutants, serving a water quality function. A DS is a grassy open channel that collects and conveys stormwater (hydrologic control), as well as allowing it to filter into the ground.

Three soil cores (15 by 5.08 cm) were taken at each BMP, one at the inlet, one at the center, and one at the outlet, using an AMS Soil Core Sampling Kit. It was necessary to remove the top layers of filtration media (pebbles) from the SSFs in order to access the soils for sampling. Soil sampling took place after a precipitation event (see Table 2 for field conditions) to allow stormwater to enter each BMP. Subsamples were removed from each core and preserved for DNA and RNA extractions. RNA subsamples were fully submerged in approximately 1 ml of RNAlater^®^. Soil bags and sealed Eppendorf tubes containing sub-samples were transported on ice. Eppendorf tubes were then placed in a −20 °C freezer prior to transportation to an ultra-low freezer (−80 °C) 2 days later.

**Table 2:** Sampling field conditions

Sampling date	15 July 2015	21 August 2015	30 September 2015
Precipitation (inches)	0.15	0.4	2.59
Temperature	31 °C	30 °C	25 °C
BMPs sampled	DP 4, DS 1, BF 2, SSF 2	DP 1, DP 2, SSF 1, BF 1, SSF 3	BF 4, BF 3, SSF 4, ^a^DP 3

Precipitation was obtained from the closest streamgage to the sampling sites USGS streamgage 391320077185901 at Slidell, MD (https://waterdata.usgs.gov/md/nwis/uv/?site_no=391328077185901&PARAmeter_cd=00045). Precipitation reflects total rainfall from the 24 h period prior to sampling. Historic temperature measurements obtained from https://www.timeanddate.com/weather/@4351708/historic?month=7&year=2015 Measurement noted is the noon measurement on each sampling day. Samples were collected between ~9 am and 12:30 pm

*DP* Dry Pond, *DS* Dry Swale, *BF* Bioretention Facility, *SSF* Surface Sand Filter

^a^Note that DP 6 was sampled on Oct 1, 2015 due to the presence of standing water on September 30th. During that period, an additional 0.21 inches of precipitation was recorded on the streamgage. Sampling temperature for DP 6 was 14 °C on 10/01/2015

### Physiochemical Data

Soils were analyzed in duplicate for pH (10 gm), NO_3_^−^(measured as NO_x_ and reported as mg N (nitrogen) per gram (g) dry soil) (3.2 gm), water content, organic C, total C, total N (6.5–9 mg), and soil texture (reported as percent fines). Wet soils were sieved through a 2 mm sieve. pH was measured in a soil-water slurry per standard soil methods (Robertson et al. 1999). NO_3_^−^ was extracted in KCl and analyzed using an AQ2 Discrete Analyzer (SEAL Analytical) within 24 h. Soil texture analysis was completed using the Bouyoucos method (Bouyoucos 1927) and residual wet, sieved soils were dried to constant weight to assess soil moisture content. Dried soils were ground and analyzed for organic C, total C, and total N using a Flash 2000 CNH-S organic elemental analyzer (Thermo Scientific) (Hall 2020).

### DNA Extractions and PCR Amplification

DNA was extracted from samples using the FastDNA^™^ Spin Kit for Soil (MP Biomedicals) according to the manufacturer’s protocol. Two sample extractions indicated possible humic acid contamination and were treated with Microcon DNA Fast Flow Centrifugal Filter Units (Millipore) per the manufacturer’s protocol. Approximately 10 ng of DNA was used in a 20 μl reaction for PCR amplification. AmpliTaq Gold^™^ DNA Polymerase (Thermo Fisher Scientific, Waltham, Massachusetts, USA) was used for the amplification. Master mix for the 16S rRNA gene amplification was subjected to ultraviolet (UV) light (Stratagene Stratalinker^®^) prior to the addition of primers and dNTPs to eliminate possible bacterial DNA contamination. UV exposure was previously titrated to ensure no impact on enzyme activity. Universal 16S rRNA bacterial primers 27 F and 357 R (Lane 1991) targeting variable regions 1 and 2, were used for bacterial identification, and *nirK* (Flacu and R3cu), *nirS* (1 F and3R), and *nosZ* (2 F and R) gene primers (Hallin and Lindgren 1999; Braker et al. 1998; Henry et al. 2006; Rosch et al. 2002) were used for the presence/absence of denitrification genes (Appendix 1). DNA was amplified using an Applied Biosystems^™^ GeneAmp^™^ PCR System 9700 thermal cycler (Thermo Fisher Scientific, Waltham, Massachusetts, USA). Duplicate PCRs were processed for all samples for each denitrification gene. *Escherichia coli* (*E.coli*) DNA was used as a positive PCR control and no DNA was used as a negative control for all PCRs. (*Sequence data submitted to NCBI, SRA accession* PRJNA555074).

### RNA Extractions, RT-PCR, and PCR Amplification

RNA was extracted using the RNeasy^®^ Mini Kit (Qiagen) or the RNeasy^®^ PowerSoil^®^ Total RNA Kit (Qiagen). Both kits were used according to the manufacturer’s standard protocols. RNA extractions were subjected to a RT-PCR protocol using random hexamers as primers and a PTC-200 Peltier Thermal Cycler (MJ Research). The GoScript^TM^ Reverse Transcription kit (Promega), as well as the Invitrogen^™^ ThermoScript^™^ RT-PCR System for First-Strand cDNA Synthesis (Thermo Fisher Scientific) were used. RT-PCR products (cDNA) underwent a standard PCR protocol, along with an RNA control, to assess product for DNA contamination. DNA contaminated samples were treated with Ambion DNase (see above) and re-run through the RT-PCR protocol. Primers for the 16S rRNA gene (27 F and 357 R) were used for amplification and the procedure was again run on an Applied Biosystems GeneAmp PCR System 9700 Thermal cycler.

### High-Throughput Sequencing of DNA and RNA

Duplicate LH-PCRs (Length Heterogeneity PCR) were performed and fingerprinted prior to sequencing in order to select the most consistent products for sequencing (Sikaroodi and Gillevet 2012). This quality control step used fusion primers for the bacterial 16S rRNA gene (27 F and 357 R). Fusion primers contain an adapter joined to an 8 base “barcode”, as well as the appropriate primers. The reverse primer (357 R) was FAM labelled on the 5-prime end. The fingerprint was run on an ABI 3130*xl* Fluorescent Sequencer (Applied BioSystems). PCR products were selected based on the fingerprints and pooled. The pool was purified twice (to ensure elimination of primer dimers and short products) with Agencourt AMPure solution (Beckman Coulter) in preparation for sequencing. The purified product was quantified using a DTX880 Multimode Fluorescent detector (Beckman Coulter) and the correct concentration was calculated to use in emulsion PCR. We used Ion Torrent technology (Thermo Fisher Scientific) with the Personal Genome Machine (PGM) for high-throughput sequencing (Appendix 2). All emulsion PCR and sequencing steps were executed using the kits and manufacturer’s protocols for the PGM. A customized PERL script was used to “demultiplex” raw sequence data from each pooled sample and to separate the sequences into individual samples based on the barcodes used for each sample at initial PCR.

### Bioinformatics and Statistical Analysis

Quality scores (FASTQ files) for each base were filtered by the PGM Ion Torrent instrument. All reads shorter than 250 bp were removed for quality control. FASTQ files were classified into abundance tables (Hall 2020) at 0.1 and 1% relative abundance cut-offs using the Baysian Classifier of the Ribosomal Database Project (RDP11) (Cole et al. 2014) (version 11.5). The Classifier was run using the standard parameters and identifications with a bootstrap level below 0.1% were discarded. 16S rRNA abundances were normalized by the total number of reads in each sample i.e., relative abundance. Bootstrap values were set at 60% to limit the inclusion of “other” and “unknown” bacteria. All analyses except for Linear Discriminant Analysis Effect Size (LEfSe) analysis focused on the genus taxonomic level with a 0.1% relative abundance cutoff (*taxanomic data available at:* 10.5066/P9AP4AH1).

A principle coordinates analysis (PCoA) was executed using Multi-Variate Statistical Package (MVSP) software. The PCoA was used to identify potential clustering of bacterial communities among BMP types, as well as differences between DNA- and RNA- derived datasets. These differences were further investigated using LEfSe analysis, which determines the features (i.e., genera) that most likely explain differences between classes (i.e., BMP types) by predicting which features violate a null hypothesis of no difference between classes (Segata et al. 2011). A 1% relative abundance cutoff (genus taxonomic level) was used for the LEfSe analysis as 0.1% produced graphs that indicated extensive numbers of differences which were impractical to include.

Lists of known denitrifiers and denitrifiers that specifically possess the *nosZ* gene were compiled using several literature studies (Rösch et al. 2002; Scala and Kerkhof 1998; Philippot et al. 2007; Zumft and Körner 2007; Green et al. 2010; Sanford et al. 2012; Shapleigh 2013; Saarenheimo et al. 2015; Lycus et al. 2017) and used to compare with the taxonomy identified in the sampled soils. Community diversity using the RNA dataset was assessed using the Shannon Diversity Index (*H*) (Spellerberg and Fedor 2003) with results averaged by BMP type.

A PICRUSt analysis (Phylogenetic Investigation of Communities by Reconstruction of Unobserved States) (https://picrust.github.io/picrust/) using standard parameters was performed on the DNA-derived dataset to investigate the potential of bacterial communities in the sampled BMP soils to conduct denitrification. PICRUSt is a bioinformatics software package (Langille et al. 2013) that is used to predict metagenome functional (gene) content from taxonomic identification markers based on the 16S rRNA gene. Distance comparisons are made between known close relatives from a reference database and organisms found in the samples. The pipeline also corrects for gene copy number by normalizing operational taxonomic units (OTUs) by the operon copy number of both known and predicted OTUs. Gene content predictions are based on protein-coding genes found in 16S rRNA gene copy numbers, while functional predictions (Clusters of Orthologous Groups or COGs) are based on the Kyoto Encyclopedia of Genes and Genomes (KEGG Orthology). This approach allowed for inference of a N cycle functional profile of the bacterial community based on the 16S rRNA gene. The PICRUSt prediction is supported by known denitrifiers identified in the samples (literature review), sequencing of the 16S rRNA gene, and the presence of specific denitrifier genes (assessed using extracted DNA).

Kruskal–Wallis, run in R, was selected as a non-parametric statistical test for data that is not normally distributed and does not fit the assumptions for parametric testing (Leon 1998). We tested transformations and found that log_10_, log_*e*_ and square root transformations did not correct the skew for all variables. To investigate further, a Dunn’s test (Dunn 1964) was run to identify which BMP types were driving significant differences observed in the Kruskal–Wallis test. The Dunn’s test does have limitations, specifically it is overly conservative on Type I error and has weak statistical power. Values were adjusted by the Holm method in an attempt to take some of these limitations into account. A canonical correspondence analysis (CCA) (using MVSP software) was run on the RNA dataset and soil sample physiochemistry values to determine the environmental gradients impacting underlying bacterial communities.

## Results

### Bacterial Community Composition and Diversity in Bulk Soil

**Fig. 2:**
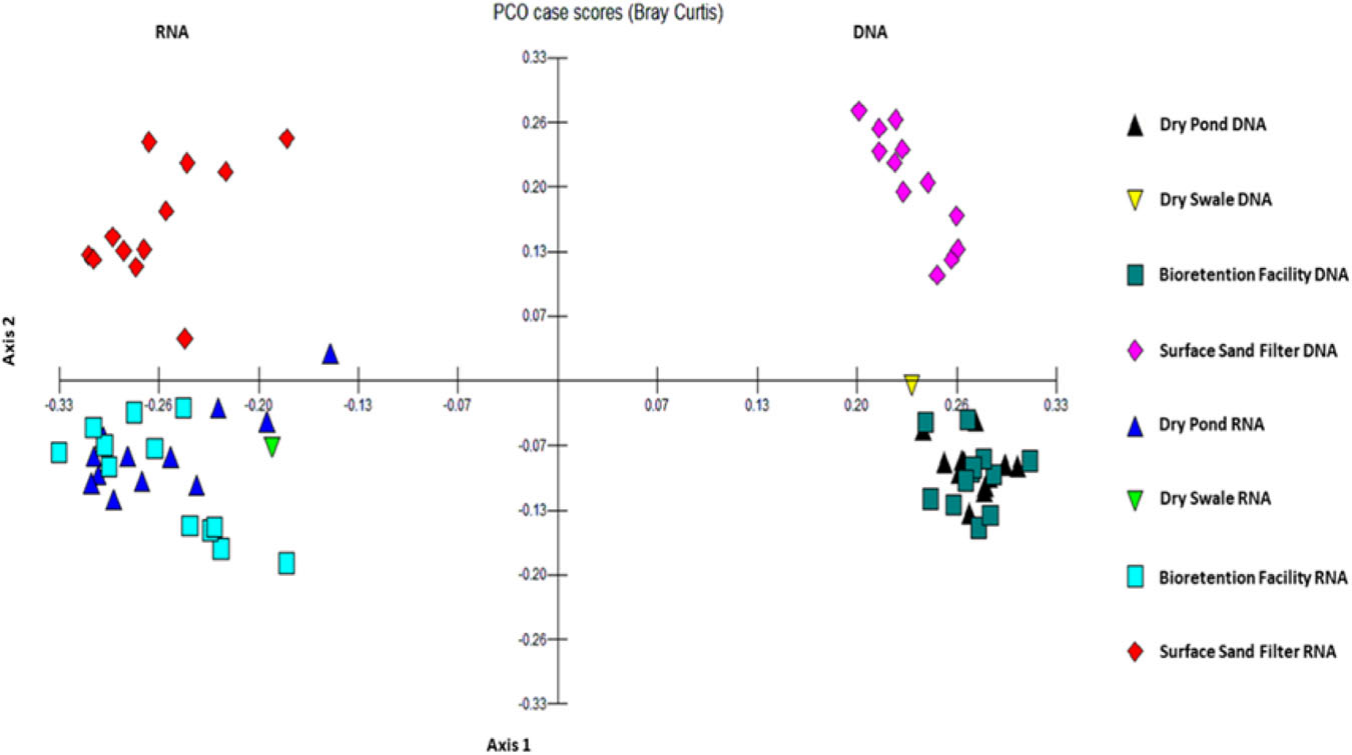
PCoA analysis of potentially active bacterial communities in four BMP types, using DNA- and RNA-derived data at the RDP11 genus taxonomic level. Clustering by BMP type was observed in both datasets. Analysis run using MVSP software

A PCoA was used to identify clustering of bacterial communities among BMP types, using data obtained from 16S gene amplification. The PCoA indicated distinct differences in the variance of DNA- and RNA-derived datasets, and among BMP types (Fig. 2). There was clustering by BMP type in the DNA-derived dataset, with some overlap between BFs and DPs, indicating similar bacterial communities by BMP type. The single DS showed a community that was somewhat similar to several BFs and DPs. There was also some clustering by BMP type in the RNA-derived dataset, but with more variance in the data, which could provide an indication of metabolic activity (Lu et al. 2009; DeAngelis et al. 2010; Blazewicz et al. 2013). There was still overlap between DPs and BFs, indicating similar bacterial communities. The single DS in the RNA-derived data had a similar community to several DPs. An additional PCoA was run on the RNA-derived dataset (Appendix 3) to examine the variance of this dataset without the influence of the DNA data. PCoA analysis of the RNA-derived data confirmed clustering by BMP type with some overlap between BFs and DPs. The single DS sample once again indicated a similar bacterial community to those of the DPs. Samples from BF 3 were clustered on the outside of the other BF samples, closer to the SSFs. Samples from DP 3 were clustered away from the other DP samples. Samples from SSF 4 (in the same treatment train as DP 3) were situated on the outer edge of the other SSF samples. Environmental variables that could be influencing potential bacterial activity are discussed in the CCA results below.

LEfSe analysis (at 1% relative abundance cutoff, Appendix 4) indicated a substantial number of differences between the DNA- and RNA-derived datasets, confirming PCoA results and providing further indication of greater variance in the RNA-derived data. LEfSe analysis using the RNA-derived dataset indicated differences among BMP types. The fewest number of differences were between BFs and DPs, indicating some similarities between bacterial communities in these BMP types. This also confirms statistical analysis that showed similarities between BFs and DPs for certain soil physiochemical parameters. The largest number of differences were between BFs and SSFs, indicating distinct differences between the bacterial communities in these two types. BFs and DPs were thus more similar to each other than BFs and SSFs or DPs and SSFs.

The sampled soils harbored high bacterial diversity of ~1000 genera and were dominated by the Proteobacteria phylum. All types of BMP sampled showed a high level of microbial diversity. Shannon Diversity Index results (Appendix 5) indicated a similar diversity index value (*H*) across all BMP types sampled, with SSFs having the highest value (*H* of 4.63) and the single DS (not averaged) the lowest value (*H* of 4.30). *H* values for BFs and DPs were 4.55 and 4.32 respectively.

**Fig. 3:**
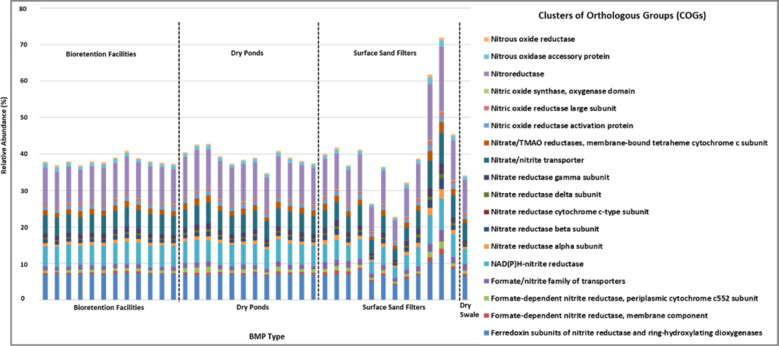
Denitrification genes as a percentage of the total COG counts of all predicted genes. Analyses are based on PICRUSt predicted COGs from16S rRNA gene DNA-derived data

PICRUSt analysis and COGs were used to predict a functional profile from the 16S rRNA-derived data, with a focus on denitrification gene content (Fig. 3). The analysis indicated similar denitrification gene predictions for all BMP types at ~0.4% of the total system community composition in each sample, including the SSFs which have a different soil chemistry than the other types (inorganic media and no vegetation). Several SSF cores had some of the highest relative abundance counts of denitrification cycle predictive genes (relative to all genes of the community). When the individual denitrification gene counts for each sample were normalized by the total denitrification gene count, all denitrification cycle sub-functions appeared in similar ratios (Appendix 6). This could be the result of various bacteria with truncated pathways conducting different parts of the denitrification cycle.

### Denitrifier Distribution by BMP Type

The 16S rRNA gene was used to identify bacterial taxa in extracted DNA and RNA. In addition, several sets of denitrification gene primers (*nirK*, *nirS*, and *nosZ*) were used to assess the presence/absence of denitrifiers in the DNA. Duplicate PCRs were obtained for all but one sample (which had no duplicate PCR product using *nirS* primers) per each gene primer set, indicating that denitrifiers were identified in all sampled BMPs, irrespective of type.

**Fig. 4:**
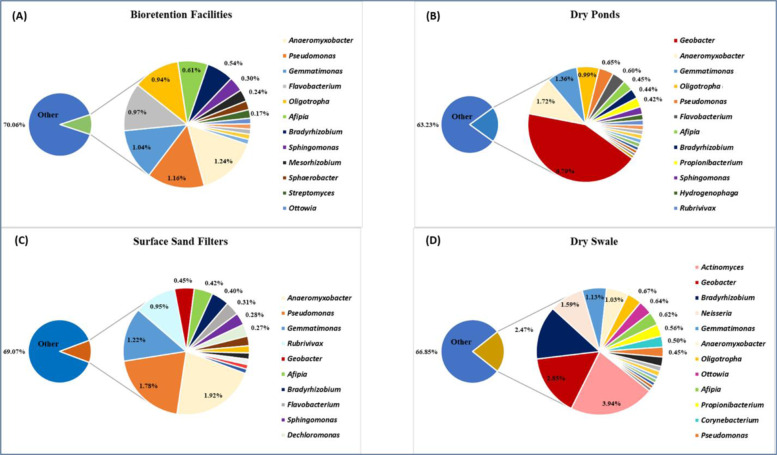
Known denitrifiers in the (**A**) Bioretention Facilities and (**B**), Dry Ponds and (**C**), Surface Sand Filters and (**D**), Dry Swale, using RNA-derived data at the RDP11 genus taxonomic level. These are relative abundances and only include genera with >0.10% abundance. General denitrifiers represent 30% or less of the overall community taxonomy per BMP type. “Other” represents the rest of the community not individually identified as denitrifier genera

The bacterial community identified in this study were dominated by the phylum Proteobacteria, along with several other phyla that are commonly found in soils (Janssen 2006; Killham and Prosser 2015), namely Actinobacteria, Gemmatimonadetes, and Bacteroidetes. The phyla Chloroflexi, Firmicutes, and Verrucomicrobia were also present, but in smaller abundances. Bacteria with the ability to denitrify are known to be common to the various subclasses of Proteobacteria (Throbäck et al. 2004). There were 45 known denitrifier genera identified in the samples (Appendix 7) based on a review of the literature, 21 of which possess the *nosZ* gene and are thus able to convert N_2_O to N_2_ gas and complete the denitrification cycle. Most abundant genera among the denitrifiers were *Geobacter* (10.19%), *Anaeromyxobacter* (5.90%), *Gemmatimonas* (4.74%), *Pseudomonas* (4.04%), *Actinomyces* (3.94%), *Bradyrhizobium* (3.85%), *Oligotropha* (2.77%), and *Afipia* (2.10%) (Fig. 4). Denitrifiers represented 30% or less of the overall community taxonomy per BMP type. There were several similarities between SSFs and BFs with regard to the presence and abundance of certain denitrifiers, *viz*. *Anaeromyxobacter*, *Pseudomonas*, and *Gemmatimonas*. This was unexpected as SSFs and BFs contain vastly different media and soils, i.e., the former has inorganic media (sand, pebbles), while the latter contains media rich in organic matter. DPs had a dominant denitrifier (*Geobacter* 6.79%) and the highest abundance in the single DS sample was for *Actinomyces* (3.94%), which was present in very low abundances of <0.1% in the other BMP types. The DS also contained a relatively high abundance of *Geobacter* (2.85%). *Bradyrhizobium* had a higher abundance (2.47%) in the DS relative to its abundance in the other BMP types.

**Fig. 5:**
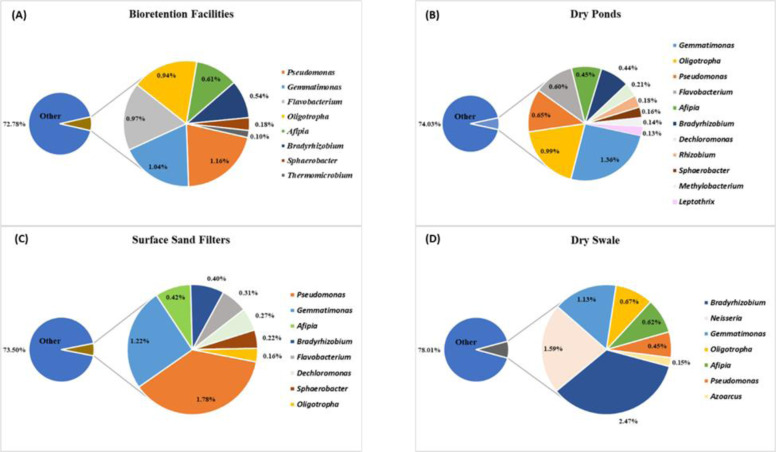
Genera known as denitrifiers that also contain the *nosZ* gene in the (**A**) Bioretention Facilities and (**B**), Dry Ponds and (**C**), Surface Sand Filters and (**D**), Dry Swale, using RNA-derived data at the RDP11 genus taxonomic level. These are relative abundances and only include genera with >0.10% abundance. Denitrifiers with the *nosZ* gene represent less than 30% of the overall community taxonomy per BMP type. “Other” represents the rest of the community not individually identified as denitrifier genera that also contain the *nosZ* gene

Most abundant genera among the denitrifiers with the *nosZ* gene were *Pseudomonas*, *Bradyrhizobium*, *Gemmatimonas*, *Oligotropha*, and *Afipia* (Fig. 5). These denitrifier communities were most similar in the BFs and DPs. Both the SSFs and the DS had a dominant *nosZ* denitrifier i.e., *Pseudomonas* (SSFs 1.78%) and *Bradyrhizobium* (DS 2.47%). Overall, there were similarities across all BMP types with regard to *nosZ* communities, based on the presence and abundances of certain genera.

### Soil Physiochemical Properties

Sampled soils were analyzed for pH, NO_3_^−^ (NO_x_), water content, soil texture (percent fines), organic C, total C, and total N (Hall 2020). The complete values are attached as Appendix 8. Histograms of the full range of soil physiochemical values across BMP types indicated that all parameters exhibited a non-normal distribution. Boxplots of the data (Appendix 9) provided trends by BMP type which were validated by the Kruskal–Wallis ANOVA test and a subsequent Dunn’s test. Kruskal–Wallis tests indicated that other than pH (*p* = 0.8), all soil physiochemical properties examined were significantly different from each other by BMP type, with a *p* (alpha) value of <0.05. A Dunn’s test (pairwise comparison) for pH confirmed that all three BMP types had similar pH (*p* > 0.05). Kruskal–Wallis analysis indicated significant differences among BMP types for NO_3_^−^, despite what appeared to be some similarity in the data values between BFs and DPs when presented graphically in a boxplot. Dunn’s test results however, indicated that BFs and DPs were not significantly different for NO_3_^−^ (adjusted *p* value of 0.37), while BFs and SSFs, as well as DPs and SSFs, were different from each other with regard to NO_3_^−^.

The Dunn’s test also showed significant differences for organic C, total C, total N, soil moisture, and percent fines among the BMP types at *p* < 0.05. This confirmed Kruskal–Wallis test results, as well as graphical representation of the data. Water content, organic C, total C, and total N were highest in BFs and lowest in SSFs. Percentage fines was highest in the DPs and lowest in the SSFs.

### Impact of Environmental Variables

**Fig. 6:**
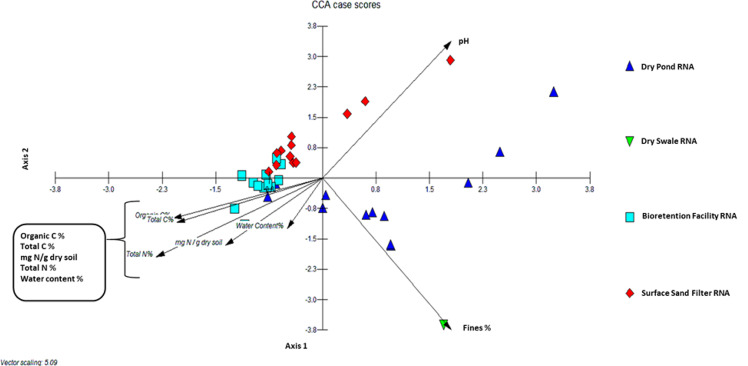
CCA analysis, run in MVSP, of the RNA-derived data at the RDP11 genus taxonomic level. Percent silt and percent clay were combined into percent fines. Results indicate clustering by BMP type and soil chemistry, with some overlap between bioretention facilities and surface sand filters

A CCA was run on the RNA-derived data to investigate potential functionality of the bacterial community (since DNA does not assume viability or activity) and to identify the primary soil parameters driving communities in the sampled BMPs (Fig. 6). Note that the soil texture measurement is constituted by three measurements, *viz*. percent sand, percent silt, and percent clay. As each of these is given equal weight in a CCA, potentially presenting a collinearity concern, percent silt and percent clay were combined into percent fines and used in the CCA as the sole measure of soil texture. Results indicated clustering by BMP type, with some overlap between BFs and SSFs. Bacterial communities in all BMP types were impacted by the surrounding soil chemistry. Communities in BFs were correlated with soil moisture, NO_3_^−^, organic C, total C, and total N. Most of the DP taxa were clustered away from the other BMP types, except for a sample from DP 2 and all three samples from DP 4, which were situated closer to the BFs and the correlation with C, N, and soil moisture. The other DPs were correlated with percent fines (soil texture), except for all samples from DP 3, which were clustered away from the other DP samples between the vectors for percent fines and pH. All three samples from SSF 4 were correlated with pH. The bulk of the other SSF taxa were more tightly clustered, with some overlap with the BFs, and were correlated with percent sand. The single DS core was separated from the other BMP types, but was closest to the DPs, with the taxa most strongly related to percent fines.

## Discussion

### Diverse Microbial Communities and Denitrifiers Identified

The sampled BMP soils harbored bacterial diversity of over 1000 genera, including an active community of taxa with the potential for denitrification. Bacterial communities clustered predominantly by BMP type, with some overlap between the BFs and DPs. The clustering indicates that each BMP type had a different bacterial community composition. This corresponds with LEfSe analyses of the RNA-derived data where BFs and DPs had fewer differences between them than between either of those BMP types and SSFs. BFs and DPs are more similar as regards environmental conditions such as plant life. Diversity index values (Shannon’s *H*) of the RNA-derived dataset indicated high diversity levels across all four BMP types. These index values were also similar across all BMP types sampled, ranging from *H* 4.30 to *H* 4.63.

PICRUSt analysis of extracted DNA indicated that N cycling contributed to the potential functions of soil bacterial communities in the sampled soils. The analysis further revealed that the potential denitrification ability would likely be similar across all BMP types, including the SSFs, despite their inorganic media content (denitrifiers are heterotrophs and require an organic C source). This could be attributed to organic matter present in stormwater runoff and moving along the treatment train, thus providing bacteria in SSFs with optimized conditions for denitrification. Organic C could also be adsorbing to the inorganic media in SSFs, creating microlayers where nitrogen cycling can occur (Perryman et al. 2011). Carbon and nitrogen content are known to impact denitrifier communities (Murray et al. 1989; Wallenstein et al. 2006; Barrett et al. 2016).

Denitrifiers were of particular interest in this study due to the legacy (previous fertilization) of arable land in the sampling area, as well as continued lawn fertilization in the urbanized watershed. Nitrogen pollution can be transported into receiving water bodies via stormwater runoff. An extensive group of diverse bacteria have the ability to denitrify, but many do not possess the complete suite of enzymes necessary for the entire process. Denitrification is thus considered a community function, with various bacteria completing different parts of the process (Wallenstein et al. 2006). This project utilized primers for three specific denitrification genes, *viz*. *nirK*, *nirS*, and *nosZ* to identify denitrifiers in the sampled soils. All three selected denitrification genes were shown to be present in all sampled BMP types in both DNA and RNA soil extractions, indicating that denitrification activity is possible under certain conditions.

There was also a large group of known denitrifier genera identified in the samples, several of these are known to carry the *nosZ* gene. The soils and filtration media of the sampled BMPs thus contained a bacterial denitrifier community that had genera in common with more typical soils found elsewhere. This could be indicative of the stormwater feeding into these BMPs, transporting and delivering bacteria, but also providing the organisms with food and energy sources.

### Association between Physiochemical Properties and Bacterial Community Profiles

Environmental factors can have an influence on the abundances and distribution of bacterial communities in general, and denitrifiers in particular (Tiedje et al. 1982; Wallenstein et al. 2006; Fierer et al. 2003; Groffman et al. 2006). A CCA was utilized to determine any significant associations between specific soil physiochemical properties and soil bacterial community patterns. The bacterial communities of the three different BMP types (the DS was not included) were found to correlate with different soil physiochemical properties. pH correlated with the communities in all three samples from SSF 4. DP 3 and SSF 4 have a higher degree of impervious cover (59–61%) than many of the other BMPs (only BF 3 at 63% has more impervious cover). This may impact soil physiochemistry and thus the bacterial communities in each affected BMP. Percent fines correlated with the bacterial community in the DS and several of the DPs. Soil moisture, NO_3_^−^, organic C, total C, and total N influenced the community in BFs.

Kruskal–Wallis ANOVA results indicated significant differences in most of the soil physiochemical properties by BMP type, with only pH having a similar median in all BMP types (*p* value of 0.8). The effect of pH on denitrification and denitrifier communities is not clear (Šimek et al. 2002) as is evidenced by the varying results of several studies. pH has been reported to be a dominant factor in denitrification gene abundance (Ligi et al. 2014), while no pH effect was observed on denitrifier gene abundances in another study (Kandeler et al. 2006). No strong relationship between soil pH and microbial activity was reported by Enwall et al. (2005), but other studies have indicated that pH has a potentially strong effect on denitrifiers in soils (Priemé et al. 2002; Deiglmayr et al. 2004; Brenzinger et al. 2015). Low pH is believed to lower denitrification rates, as well as influence the ratio of N_2_O to N_2_ gas (Brenziner et al. 2015; Šimek and Cooper 2002; Liu et al. 2010), but this effect could be minimized by the ability of certain acid-tolerant denitrifiers to denitrify at low pH. Different denitrifiers can thus perform similar functions in different environmental conditions (Brenzinger et al. 2015). Low denitrification rates in acidic conditions could also be attributed to limited organic C and nitrogen rather than the direct influence of low pH (Šimek and Cooper 2002). In general, the bacterial communities in BFs and DPs were more similar to each other than to those in the SSFs with regard to soil chemistry. This is likely influenced by their similarities in filtration media and plant life.

A Dunn’s test confirmed Kruskal–Wallis results that most of the physiochemical parameters were significantly different per each BMP type. pH was not significantly different among the BMP types. Dunn’s test results also indicated that BFs and DPs were not significantly different for NO_3_^−^, although SSFs were significantly different from both BFs and DPs for NO_3_^−^. This could indicate that not all physiochemical properties are equally important for bacterial community composition and functionality in these sampled BMP soils.

## Conclusion

Urbanization and associated land use change can result in an increase in stormwater runoff and a host of repercussions for receiving water bodies, including eutrophication from excess nitrogen inputs. Urban stormwater BMPs are mitigation measures to reduce the volume of stormwater and its rate of movement through the watershed. The aims of this observational study were to examine the bacterial communities in the soils of selected BMP types (using the 16S rRNA gene from extracted DNA and RNA) to assess whether there were different bacterial communities by BMP type, whether these communities were impacted by the surrounding soil physiochemistry, and whether denitrification was possible in these BMP soils.

The soils of sampled BMPs indicated a varied and rich bacterial community of more than 1000 genera. This included a community of known denitrifiers that were found in all BMP types sampled and which clustered predominantly by BMP type. While emphasis was placed on denitrifiers that carried specific denitrification genes (*nirK*, *nirS*, and *nosZ*), it should be noted that there is recent evidence to indicate the presence of two *nosZ* clades (Sanford et al. 2012; Yoon et al. 2016). Clade I contains known denitrifiers that harbor an additional denitrification gene, such as *nirS*, in addition to *nosZ (*for example, *Pseudomonas aeruginosa*). Clade II consists of organisms that have the *nosZ* gene, but no other denitrification genes (for example, *Wolinella succinogenes*). Yet they are able to remove N_2_O from the environment and serve as N sinks. The use of the *nosZ* gene as an indicator of denitrification is thus not as simplistic as it once seemed. Future work will need to take this second clade into account and a decision taken whether to include Clade II *nosZ* organisms as denitrifiers. Although this distinction was not addressed in this study, several Clade II *nosZ* bacteria were identified in the sampled soils.

The bacterial community in the sampled BMPs was correlated with various soil parameters, which may have impacted the community composition in each BMP type. While several of these parameters are known to provide favorable conditions for denitrification, in this study, it appears that the impact of pH on potential denitrification and denitrifier communities in BMPs requires further investigation. Statistical analysis of pH measurements indicated similarities across the three BMP types.

Based on the presence of a robust, diverse, metabolically-active bacterial community that includes a number of known denitrifiers, the presence of denitrification genes, the assumption of bacterial viability (using extracted RNA), and suitable conditions for denitrification, it is possible that denitrification can occur in all BMP types sampled. No inference is made, however, regarding efficiency of the process. Future work with regard to the assessment of BMP types for denitrification potential might therefore include SSFs, despite their mineral media content. Tiedje et al. (1982) identified denitrifying enzymes in sandy soil (the primary soil texture in SSFs), despite low (20%) O_2_ levels in the well-drained soils. This was attributed to the presence of high levels of organic C which can support higher populations of heterotrophs. Our results indicated that BMP soil C content influenced bacterial communities in the sampled BMPs. Stormwater can carry a variety of substrates and the presence of sufficient C and nutrient loads, combined with the alternating wet/dry cycles available in many stormwater BMPs, could promote conditions for bacterial growth in receiving BMPs, irrespective of BMP type.

The findings of this study highlight the role that certain types of stormwater BMP could play in removing pollutants such as nitrogen from stormwater runoff before it enters receiving water bodies and causes algal growth and eutrophication both locally and downstream. This includes BMP types not specifically designed for denitrification or containing organic-rich soils and filtration media. This would be especially important for land managers in watersheds with legacy nitrogen from former agricultural land use.

## Disclaimers

Any use of trade, firm, or product names is for descriptive purposes only and does not imply endorsement by the U.S. Government.

## Data Availability

Soil chemistry and microbial taxonomy data available at: 10.5066/P9AP4AH1; Sequence data submitted to NCBI, SRA accession PRJNA555074.

## Appendix 1 PCR primers used

**Table:**

Primers	Targeted extractions	Sequence	Reference
*Universal bacterial*			
27 F	DNA and RNA	5′-AGAGTTTGATCMTGGCTCAG-3′	Lane 1991
357 R	DNA and RNA	5′-GCTGCCTCCCGTAGGAGT-3′	Lane 1991
*Denitrification*			
nirK Flacu	DNA	5′-ATCATGGTSCTGCCGCG-3′	Hallin and Lindgren 1999
nirK R3cu	DNA	5′ -GCCTCGATCAGRTTGTGGTT-3′	Hallin and Lindgren 1999
nirS 1 F	DNA	5′-CCTA(C/T)TGGCCGCC(A/G)CA(A/G)T-3′	Braker et al. 1998
nirS 3 R	DNA	5′-GCCGCCGTC(A/G)TG(A/C/G)AGGAA-3′	Braker et al. 1998
nosZ 2 F	DNA	5′-CGCRACGGCAASAAGGTSMSSGT-3′	Henry et al. 2006
nosZ R	DNA	5′-CATGTGCAGNGCRTGGCAGAA-3′	Rosch et al. 2002
